# Effect of Coconut Water Concentration on Survival of Bench-Dried Periodontal Ligament Cells

**DOI:** 10.5005/jp-journals-10005-1074

**Published:** 2011-04-15

**Authors:** Sanaa Al-Haj Ali, Suhad Al-Jundi, Nizar Mhaidat, Lama Awawdeh, Randa Naffa

**Affiliations:** 1 Department of Preventive Dentistry, Jordan University of Science and Technology, Irbid, Jordan; 2 Department of Clinical Pharmacy, Jordan University of Science and Technology, Irbid, Jordan; 3 Department of Restorative Dentistry, Jordan University of Science and Technology, Irbid, Jordan; 4 Molecular Biology Research Laboratory, University of Jordan, Amman, Jordan

**Keywords:** Avulsion, Cell culture, Coconut water, Dry period.

## Abstract

**Background :** Coconut water is a biological and sterile liquid. It contains a variety of electrolytes, sugars and amino acids. The purpose of this study is to evaluate the effect of concentration and maturity of coconut water on its ability to preserve human PDL cell viability after exposure to dry time of up to 120 minutes using an *in vitro* cell culture model.

**Methods :** PDL cells were obtained from sound permanent first molars which were cultured in Dulbecco’s Modified Eagle’s Medium (DMEM). Cultures were subjected to 0, 30, 60, 90 and 120 minutes dry periods then incubated with 100 and 50% young and mature coconut water for 45 minutes at room temperature (18-26°C). Untreated cells at 0 and 120 minutes, and cells incubated in DMEM served as controls. PDL cell viability was assessed by MTT assay. Statistical analysis of data was accomplished by using one-way analysis of variance complemented by Tukey test, and the level of significance was 5% (p < 0.05).

**Results :** 100% mature coconut water (MCW) was better than 50% dilutions obtained from mature or young coconuts. However, no significant benefit to the cells was noticed from the addition of the soaking step prior to 30 minutes dry time.

**Conclusion :** Avulsed teeth which are left dry for > 30 minutes may be benefited from soaking in 100% mature coconut water; further studies on simulated avulsion in animal models are needed to verify the above results.

## INTRODUCTION

Tooth avulsion represents 0.5 to 3% of traumatic injuries to permanent teeth. It is one of the most severe injuries characterized by complete displacement of the tooth outside the socket.^1^ Treatment of avulsion usually focuses on encouraging viability and repair of the periodontal ligament (PDL).^2^ This is usually achieved by replantation within the first 5 minutes of the event (immediate replantation).^3^ However, this is not always possible under certain conditions. Therefore, the presence of a dry period, which is usually followed by placing the tooth in a liquid storage medium, is a common finding in avulsed teeth and is often unreported.^4^

The dry period is harmful, since it causes death of PDL cells^5^ and dehydration of the dental pulp.^6^ In addition, it correlat with an increased incidence of ankylosis and replacement resorption.^2,6,7^ Some reports even suggested that the increase in the dry period has a greater effect on the resorption risk than extending the soaking period in storage media.^2^ Therefore, every effort should be made to minimize the dry period and to use an optimal storage medium when immediate replantation is not feasible.

Various storage media have been investigated for their ability to preserve viability of PDL cells. Recently, coconut water was suggested as a storage medium for avulsed teeth,^8-10^ since it is a biological, hygienic and sterile, it also contains a variety of electrolytes (potassium, calcium, magnesium), sugar (glucose, fructose) and amino acids.^10^ Coconut water was found to be superior to propolis, milk and Hank’s balanced salt solution (HBSS) in preserving PDL cell viability after exposure to 30 minutes dry time.^8^ However, these studies neither indicate the concentration of coconut water used nor the maturity of the fruit used to collect coconut water from, in addition the efficacy of coconut water to maintain PDL cell viability after exposure to extended periods of dry time was not tested before, therefore, the main aim of the present study is to evaluate the effect of concentration and maturity of coconut water on its ability to preserve human PDL cell viability after exposure to dry time of up to 120 minutes using an *in vitro* cell culture model.

## MATERIALS AND METHODS

### Collection of Coconut Water

Two good quality Indian coconuts were used in this study; one relatively young and another mature coconut. Under sterile conditions, the young coconut was cut with a large sterile knife until a relatively soft flesh was exposed, then a sterile number 15 scalpel was used to cut a triangular piece through the exposed coconut flesh. While, the mature coconut was opened by twisting a sterile screw through the soft eye of the nut. Young and mature coconut water samples were obtained by a sterile syringe and filtered. In addition, 50% dilutions were prepared in Dulbecco’s Modified Eagle’s Medium (DMEM; BioWhittaker, Belgium).

### Primary Culture of Human Periodontal Ligament Cells

The design of the study was approved by the institutional review board (IRB) and the ethical committee of the faculty of medicine at Jordan University of Science and Technology. Written consent was obtained before extraction of potential teeth.

The PDL tissue explants were obtained from two fully erupted sound maxillary first molars, extracted from an 11-year-old patient, for orthodontic reasons. The teeth were extracted as atraumatically as possible after asking the child to rinse with chlorhexidine mouthwash, then the middle third of the root surface was mechanically scraped with a number 15 scalpel using aseptic techniques to obtain samples of PDL tissue. The PDL tissue was diced into small tissue explants of 1 mm^3^. The tissue explants were placed into tissue culture flasks (25 cm^2^), and they were incubated with DMEM containing glucose (4.5 gm/l), Penicillin (100 μg/ml), Streptomycin (100 μg/ml), Amphotericin B (0.25 μg/ml) and 10% heat-inactivated fetal bovine serum (all obtained from BioWhittaker, Belgium).

**Fig. 1:**
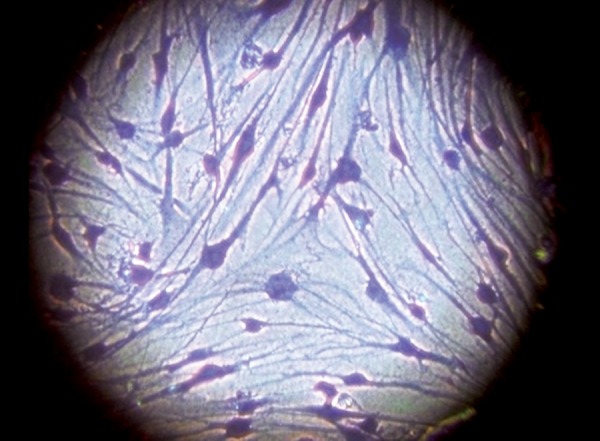
Periodontal ligament fibroblasts at 80 to 90% confluency (original magnification 100X)

Cells were grown at 37°C in a humidified atmosphere of 5% CO_2_ in air. After 4 to 5 weeks, cells reached confluence then they were detached after trypsinization (0.25% trypsin with ethylene diamine tetra-acetic acid (EDTA) obtained from Sigma-Aldrich, USA) for 5 to 10 minutes and transferred to larger flasks (75 cm^2^) for continued growth. Subconfluent cultures were characterized to assure their PDL cell phenotype by the presence of alkaline phosphatase. Culture medium was renewed, once needed, until cells reached 80 to 90% confluency (Fig. 1).

The 7th to 15th passages of PDL cells were used in the experiment. Cells were seeded at a density of 1.0 × 10^4^ cells/ well in 100 μl full growth medium in 96-well plates. The plates were incubated at 37°C in a humidified atmosphere of 5% CO_2_ in air for 48 hours.

### Exposure of PDL Cultures to Experimental Media

On the day of treatment, the culture medium was drained from each well and the cells were rinsed with phosphate buffered saline solution (PBS). Cultured cells were distributed into six experimental groups corresponding to the dry period tested.

In each experimental group, PDL cells were bench-dried to a certain period (0, 30, 60, 90 or 120 minutes) in a separate 96-well plate, then they were incubated with 200 Hl of 100% young coconut water (YCW), 50% YCW, 100% mature coconut water (MCW), 50% MCW or DMEM for 45 minutes at room temperature. Untreated cells at 0 and 120 minutes and cells soaked in DMEM served as controls. Experiments were carried out in quadruplicate wells, and they were repeated on two different occasions.

### Assessing the Viability of Cells by MTT Assay

**Fig. 2:**
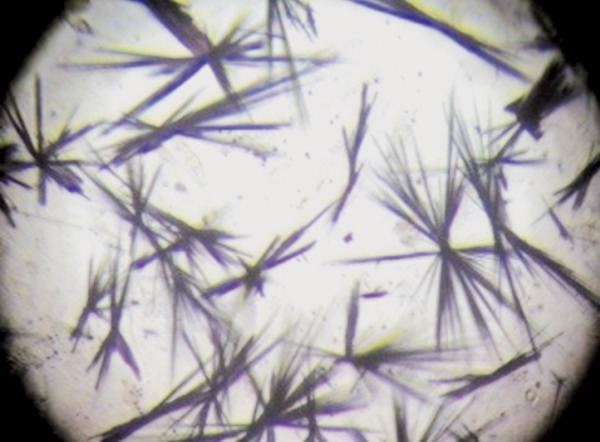
Formazan crystals (original magnification 100X)

A 5 mg/ml solution of MTT (Sigma-Aldrich, USA) in PBS was produced and sterilized by filter. After incubation with experimental media, all media were removed and the PDL cells were washed twice with PBS, then 100 μl of DMEM and 10 μl of MTT solution were added to all wells and all plates were incubated for 2 to 4 hours at 37°C. The supernatant was then eliminated, and 100 μl of DMSO solvent (Sigma, Aldrich, USA) was added to each well. The plates were then incubated for 15 to 20 minutes at 37°C (Fig. 2).

The absorbance at 570 nm was measured with a microtiter plate reader (Tecan, Austria) with absorbance at 650 nm used as a reference. The mean absorbance value of untreated wells at 0 minutes was used to indicate the 100% viability value.

The percentage of viable cells was calculated according to the following equation: % of cell viability = (mean absorbance of experimental wells/mean absorbance of untreated wells at 0 minutes) * 100%.

**Table 1:** **Table 1:** Mean absorbance, % of viability of cells in tested media and control cells at various dry periods

*Medium*		*Dry period*																			
		*0*				*30*				*60*				*90*				*120*			
		*Mean*		*%* *viability*		*Mean*		*%* *viability*		*Mean*		*%* *viability*		*Mean*		*%* *viability*		*Mean*		*%* *viability*	
100% YCW		0.75		92.87		0.43		52.58		0.15		16.18		0.0503		3.39		0.044		2.62	
50% YCW		0.81		99.65		0.49		59.89		0.10		10.3		0.03		0.42		0.02		0	
100% MCW		0.86		100.00		0.50		61.32		0.17		19.06		0.0508		3.45		0.044		2.58	
50% MCW		0.73		90.77		0.48		59.12		0.11		10.92		0.035		1.25		0.030		0.61	
DMEM		0.78		95.05		0.41		48.43		0.86		6.6		0.028		0		0.023		0	
Untreated control cells		0.81		100.00		0.67		81.65		0.13		12.12		0.05		2.23		0.042		1.06	
ANOVA p-value		< 0.001																			

### Statistical Analysis

Statistical analysis of data was accomplished by using one-way analysis of variance and complemented by Tukey test to compare all possible pairs of means within each experimental group with each other to assess the direction of the difference between the means. The level of significance was 5% (p < 0.05).

To test reproducibility/reliability, intraclass correlation coefficient based on one-way random model was used. Experiments were considered reliable since the values of α were between 0.9 and 1.000.

## RESULTS

The mean absorbance and percentage of viable cells are shown in Table 1. There was a significant drop in the mean percentage of viable cells for all tested media, as the dry period progressed from 0 to 90 minutes (p < 0.05). However, the drop was insignificant from 90 to 120 minutes.

Analysis of variance test showed a significant difference among the groups. Tukey’s honest significance to difference test showed that 100% MCW was significantly better than 100% YCW and 50% MCW at 0 minutes. At 30 and 60 minutes dry time, 100% MCW was significantly better than DMEM and insignificantly different from other concentrations of coconut water tested. At 90 and 120 minutes dry times, 100% MCW and 100% YCW became significantly better than all other experimental media. Untreated control cells showed significantly better viability scores in the first 30 minutes of dryness than cells soaked in the tested media, thereafter, soaking cells in 100% coconut water gave significantly better viability scores than control cells, however, the difference from untreated cells at 120 minutes was insignificant.

**Fig. 3:**
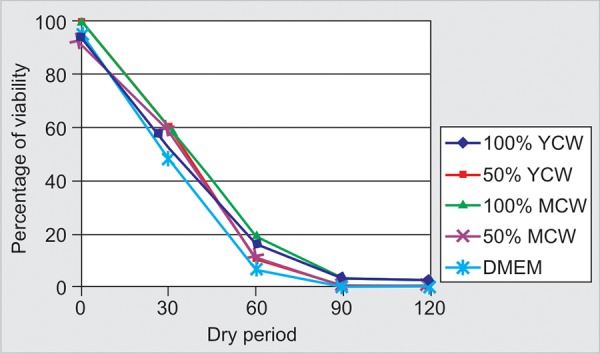
Periodontal ligament cell viability in the tested media at various periods of dry time

Among experimental media, 100% MCW resulted in the best percentage of viable cells at all dry periods (Fig. 3).

## DISCUSSION

Avulsion is one of the most severe dental injuries, studies in this field focus on transferring injured teeth in optimal media that reduce the damage and enhance viable PDL cells. The results of the present study showed that beyond 30 minutes of dry time, PDL ligament cells benefited from soaking in 100% coconut water.

Management of avulsion focuses on replantation at the earliest time possible to avoid any extra damage to the PDL or infection of the pulp.^11^ Therefore, the single most important factor to ensure a favorable outcome after replantation is the speed with which the tooth is replanted,^12^ or the extra-alveolar period. The extra-alveolar period is usually a combination of dry and wet periods.^13^ It has been shown that when the dry period exceeds 15 minutes, the number of surviving cells will be limited in both number and function following their transfer to a liquid storage medium.^14^ Our study showed that drying periodontal ligament cells resulted in a significant drop in the number of viable cells up to 90 minutes dryness, beyond which the number of viable cells became negligible. Optimal storage media tend to prolong the viability of the remaining PDL cells at the root surface^4^ leading to slowing down of the ongoing and inevitable process of cellular damage that occurs in the PDL of the avulsed tooth.^15^

In the dental literature, many methods were used to collect and quantify PDL cells, such as enzymatic disaggregation procedures that involved trypsinization,^16,17^ treatment with dispase and collagenase^8,10,19^ or collagenase and protease^9^. In addition, several studies used manual staining methods, such as trypan blue exclusion,^8,10,18-20^ neutral red^16^ and fluorescein diacetate to quantify viable cells.^17^ The disadvantages of enzymatic disaggregation procedures are the damages that may occur to some cells or the incomplete disaggregation.^21^ Whereas, manual staining methods of viable cells may not give any idea about the actual physiologic health or metabolic capabilities of the cell which are likely critical for the prevention of resorption squealae postreplantation.^19^

In this study, the number of viable cells was calculated by means of a highly calibrated and reliable microtiter plate reader. In addition, we used MTT assay which is a widely used assay to measure viability with the advantages of being sensitive (detecting as few as 10^3^ viable cells/ml) and accurate, with color development strongly correlating with cell numbers.^22^ Cell culture model was selected for this study to ensure standardization and reproducibility of the study.

Coconut water was recently suggested as a storage medium due to its availability in some parts of the world.^8-10^ One study used a collagenase-dispase assay to investigate the potential of coconut water in maintaining viable periodontal ligament cells on simulated avulsed teeth, compared to HBSS and milk, and found that coconut water kept significantly more PDL cells viable.^10^ Another study used the same method to compare the efficacy of coconut water with propolis, HBSS and milk in maintaining viable periodontal ligament cells and found that coconut water had significantly more PDL cells viable compared with propolis, HBSS and milk.^8^ A recent study using cell culture model demonstrated that coconut water may be a better alternative to HBSS and milk, in terms of maintaining cell viability.^9^

This study demonstrated that full concentration of coconut water was better than 50% dilutions, however, no significant improvement from the addition of the soaking step was found before 30 minutes of dry time. At 0 to 30 minutes, all experimental media were significantly worse than untreated control cells. This finding is in line with Gopikrishana et al,^10^ who found that after 30 minutes dry time, coconut water was significantly worse than the positive control, which was teeth assessed immediately with no soaking.

It can be hypothesized from the findings of this study that soaking in 100% coconut water could maintain the viability of healthy cells. However, the ability to revitalize damaged cells or to stimulate the remaining cells was unlikely under the conditions of this study. This hypothesis could be true, if the high osmolarity of coconut water was considered (372 mOsm/L).

## CONCLUSION

The study showed that full concentration of coconut water was better than 50% dilution, and mature fruits were better to obtain the coconut water from. The findings of this study also imply that avulsed teeth which are left dry for > 30 minutes may benefit from soaking for 45 minutes in 100% coconut water. However, further research on clinically simulated avulsion in animal models is needed to validate the results of this *in vitro* study.
